# A Leaf-Colour- and Shadow-Structure-Guided Method for Correcting Wheat Canopy Reflectance

**DOI:** 10.3390/s26206548

**Published:** 2026-10-15

**Authors:** Hongfeng Yu, Hongyan Gao, Peng Wang, Zhaoyu Zhai, Wei Qiu, Yongqian Ding

**Affiliations:** 1 College of Engineering, Nanjing Agricultural University, Nanjing 210031, China; hongfengyu@njau.edu.cn (H.Y.); gaohongyan@stu.njau.edu.cn (H.G.); 2 College of Smart Agriculture (College of Artificial Intelligence), Nanjing Agricultural University, Nanjing 210031, China; 2024819033@stu.njau.edu.cn (P.W.); zhaoyu.zhai@njau.edu.cn (Z.Z.)

**Keywords:** wheat canopy, shadow interference, reflectance correction, Blue Deficient Index (BDI)

## Abstract

**Highlights:**

**What are the main findings?**

- A leaf-colour- and shadow-structure-guided framework was developed to correct wheat canopy RGB reflectance under natural illumination by combining BDI-derived shadow grades with RGB, HSV, and Lab colour features.
- The Fullgrade_noflat representation combined with 1D-CNN obtained optimal correction performance with test R^2^ of 0.8773, RMSE of 0.0213 and MAE of 0.0161.

**What are the implications of the main findings?**

- The proposed approach reduced the within-day CVs of plot-level RGB observations, while the plotted trends across sampling dates remained visible.
- It outperformed BDI-Otsu, RGB-Y-Otsu and SDI local correction methods in terms of RMSE, MAE and CV metrics.

**Abstract:**

Variation in illumination and canopy geometry can affect the comparability of RGB measurements acquired at different times of day. This study proposes a wheat canopy reflectance correction method guided by leaf-colour and shadow-structure features. UAV RGB images were acquired under different nitrogen rates, sampling dates and acquisition times. SP, HSV, Lab and BDI features were extracted to build four input schemes, which were fed into multiple neural networks for correction. The Fullgrade_noflat input, which retains the BDI grade order, combined with 1D-CNN gave the most accurate predictions of the plot-level daily mean RGB reference among the tested configurations (R^2^ = 0.8773, RMSE = 0.0213 and MAE = 0.0161). The within-day CV decreased for all three RGB channels. The model also yielded lower errors than three RGB shadow-compensation implementations evaluated on the same dataset. These results indicate improved within-day consistency under the conditions studied; performance in other environments and effects on agronomic trait analysis require further evaluation.

## 1. Introduction

Wheat is one of the world’s major staple crops, and rapid, non-destructive monitoring of its growth status is essential for precision agriculture and yield prediction. Field imaging technologies based on unmanned aerial vehicles (UAVs) or fixed platforms, particularly RGB imaging, have become major tools for high-throughput crop phenotyping because of their low cost, high spatial resolution, and ease of acquisition [1,2].

However, under natural illumination, complex coupling occurs between the three-dimensional canopy structure, including plant height, row spacing, and leaf orientation, and the solar illumination geometry, including solar elevation and azimuth angles. This coupling results in pronounced local illumination heterogeneity in the acquired RGB images. This heterogeneity is mainly reflected in two aspects. First, shadow effects arise from mutual occlusion among leaves and shadows cast by plants, causing leaves with similar physiological properties to show inconsistent brightness and colour in RGB images. Second, illumination gradients result from uneven light interception across upper and lower canopy layers and within and between crop rows, producing gradual transitions from illuminated to shaded regions. Moreover, the bidirectional reflectance characteristics of leaves, which vary with the viewing–illumination geometry, further increase the non-stationarity of reflectance signals among pixels [3]. Consequently, canopy RGB values reflect both plant characteristics and illumination geometry [4]. Variation among plots may also reflect cultivar, nitrogen treatment and canopy structure. Accordingly, within-day variation was evaluated relative to the daily reference of each plot [5].

Correction methods differ in their data requirements, targets and output scales, and can be grouped here into four categories: (1) physical model-based, (2) radiometric or image-statistical, (3) shadow detection and compensation, and (4) deep learning-based methods. Physical model-based correction methods, such as the Scattering by Arbitrarily Inclined Leaves (SAIL) model and the coupled PROSPECT + SAIL (PROSAIL) model, explain variations in canopy reflectance in terms of leaf optical properties, canopy structure, and sun–sensor geometry. However, they generally require comprehensive structural parameters, such as leaf area index and mean leaf inclination angle, as well as synchronized multi-angular observation geometry. These requirements make parameter acquisition complex and limit their direct applicability to high-throughput RGB image correction under field conditions [6,7]. Radiometric or image-statistical correction methods, such as empirical line correction, reference panel-based correction, and Retinex-based methods, can correct variations in overall irradiance or improve image brightness distribution. However, their ability to adapt to local and continuous illumination gradients within the canopy, which are caused by leaf occlusion and inter-row shadows, remains limited [8,9]. Shadow detection and compensation methods can apply targeted corrections to shaded regions, but their effectiveness depends strongly on the accuracy of shadow segmentation. In canopy images containing dark-green leaves, partial shadows, and deep shadows simultaneously, a simple binary classification of shaded and non-shaded regions cannot adequately characterize continuous brightness variations, resulting in unnatural illumination transitions in the corrected images [10,11]. Deep learning-based correction methods are capable of learning complex nonlinear mappings, but they typically require large training datasets. The training targets and output scales of these approaches also differ from the plot-level daily reference used here [12].

To address these limitations, a key premise is that reflectance fluctuations in canopy RGB images essentially arise from the interaction between biological variation in leaf colour and changes in illumination–shadow structure. To achieve effective reflectance correction, it is essential that the input features simultaneously characterize leaf-colour properties and distinguish shadow gradients. Accordingly, this study selected multidimensional and physically interpretable feature parameters as model inputs to support robust reflectance correction. Among these features, central-tendency and distribution-shape parameters derived from the RGB colour space can quantitatively describe the intensity, distributional bias, and concentration of canopy leaf colour. Hue–Saturation–Value (HSV)-derived colour parameters are relatively insensitive to geometric transformations and remain robust under varying illumination conditions [13]. The Lab colour space separates the lightness component (L) and further characterizes canopy colour variation through the red–green channel (a) and yellow–blue channel (b), and has been shown to be suitable for identifying wheat phenotypic status [14]. The Blue Deficient Index (BDI) was used to organize canopy pixels into ordered grades based on differences among the RGB channels [15]. Therefore, we propose integrating the above leaf-colour features with BDI-derived shadow-structure features to construct a feature representation framework that preserves the ordering of BDI grades within each image. This integration is essential for overcoming the limitations of existing methods and achieving reflectance correction with low parameter dependence and high fidelity.

Accordingly, this study uses leaf-colour and BDI-grade features to predict the plot-level daily mean RGB reference from a single wheat canopy image. The method combines leaf-colour features with ordered BDI-grade features to represent variation within each canopy image. On this basis, to fully capture the nonlinear correction patterns embedded in the multidimensional feature vectors, this study compared and tested the performance of five machine learning algorithms for reflectance correction: multilayer perceptron (MLP), Kolmogorov–Arnold Network (KAN), back-propagation neural network (BPNN), long short-term memory network (LSTM), and one-dimensional convolutional neural network (1D-CNN). Their prediction performance was evaluated against the plot-specific daily reference. The main contributions of this study are threefold. First, we propose a feature representation framework that integrates canopy leaf-colour and shadow-structure features, preserving the order of the BDI grades. Second, based on this framework, we systematically constructed and evaluated different feature combinations and machine learning algorithms for reflectance correction, thereby validating the effectiveness of the proposed method. Third, the predicted plot-level RGB reflectance values showed lower within-day CVs in the current dataset. Pixel values provided the input features, and the model produced three plot-level RGB reflectance values.

## 2. Materials and Methods

### 2.1. Experimental Design and Field Data Collection

**Figure 1:**
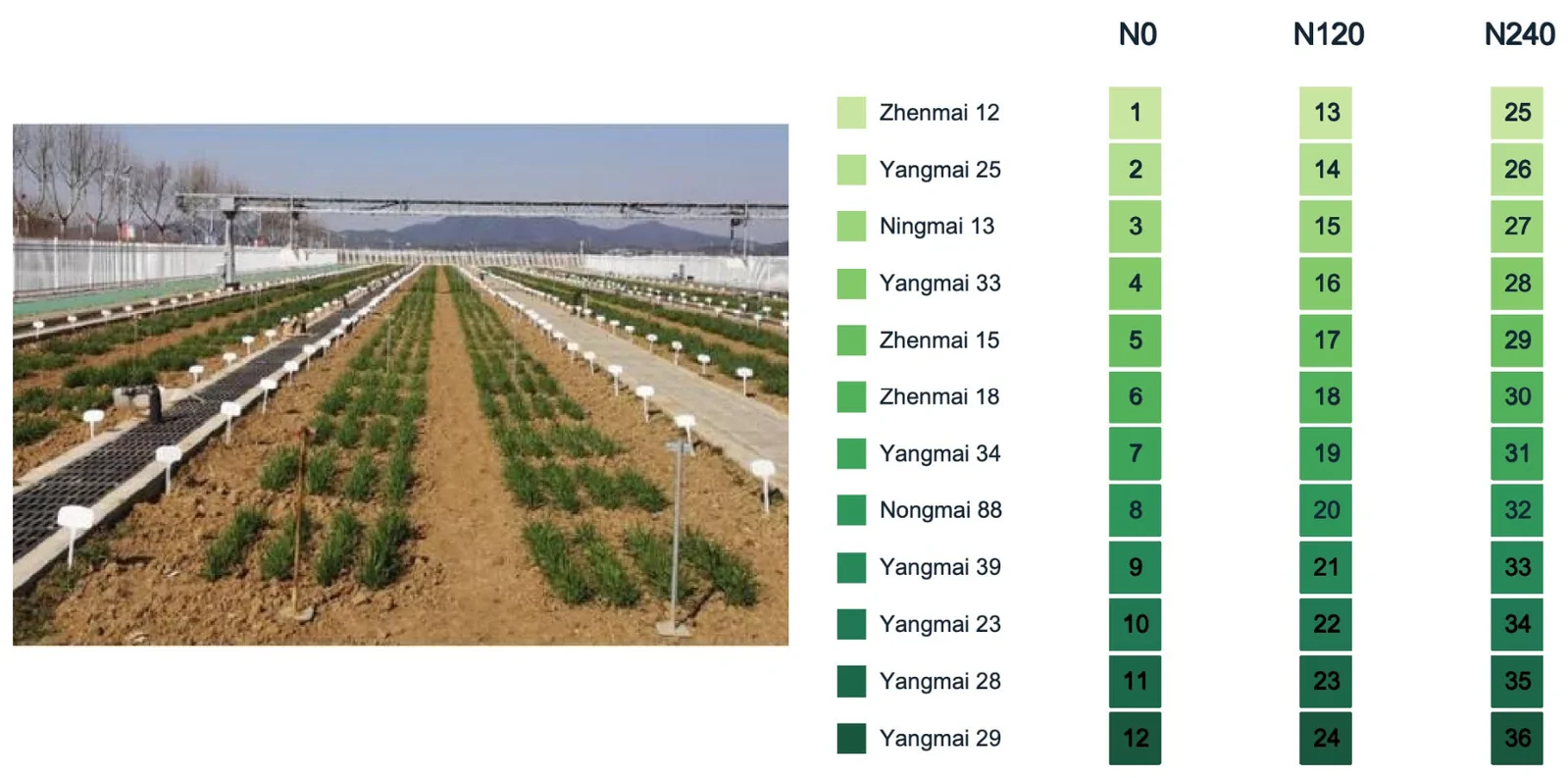
Field experimental setup for wheat canopy image acquisition.

The wheat canopy image acquisition experiment was conducted at the Baima Experimental Base of Nanjing Agricultural University in Nanjing, Jiangsu Province, China. The planting time for the experimental plots in this study was in early December 2024. Different fertilization levels and wheat varieties were set up to generate diverse canopy structures. The plot layout is shown in Figure 1. Twelve wheat cultivars (Zhenmai 12, Yangmai 25, Ningmai 13, Yangmai 33, Zhenmai 15, Zhenmai 18, Yangmai 34, Nongmai 88, Yangmai 39, Yangmai 23, Yangmai 28 and Yangmai 29) were grown under three nitrogen treatments (N0, N120 and N240), with one independent plot for each cultivar–nitrogen combination, giving 36 plots. Plots 1–12, 13–24 and 25–36 corresponded to N0, N120 and N240, respectively; each group included one plot of each cultivar. Each plot contained four wheat rows and measured approximately 1.0 m × 0.8 m, with a row spacing of 0.25 m.

The plot was the experimental unit; repeated images of the same plot were temporal observations. Field images were acquired using a DJI M3M unmanned aerial vehicle (UAV; DJI, Nanshan District, Shenzhen, China). During each image acquisition batch, a corresponding white reference image was acquired and paired with the canopy images collected within the same batch. The UAV acquired images along a preprogrammed flight route at predetermined times and locations, with the flight altitude and speed maintained at 3.5 m and 5.0 m s^−1^, respectively. Throughout image acquisition, the camera lens was oriented vertically downward, and the ISO was set to 100. The acquired RGB images had a resolution of 5280 × 3956 pixels and were saved in JPG format.

Wheat canopy images were collected on six sunny sampling dates from 25 March to 29 April 2025, with 6–8 days between consecutive dates. On each date, each of the 36 plots was imaged every 30 min from 09:00 to 15:30. All 14 observations per plot and date were included, giving 36 × 6 × 14 = 3024 plot-level canopy images. Throughout image acquisition, an illuminance meter, solar elevation angle observation software, and a standard white reference panel were used to record the solar elevation angle, light intensity, and white reference data. The specifications of the experimental equipment used in this study are summarized in Table 1.

### 2.2. Construction of the Dataset

#### Preprocessing of Wheat Canopy Images

**Figure 2:**
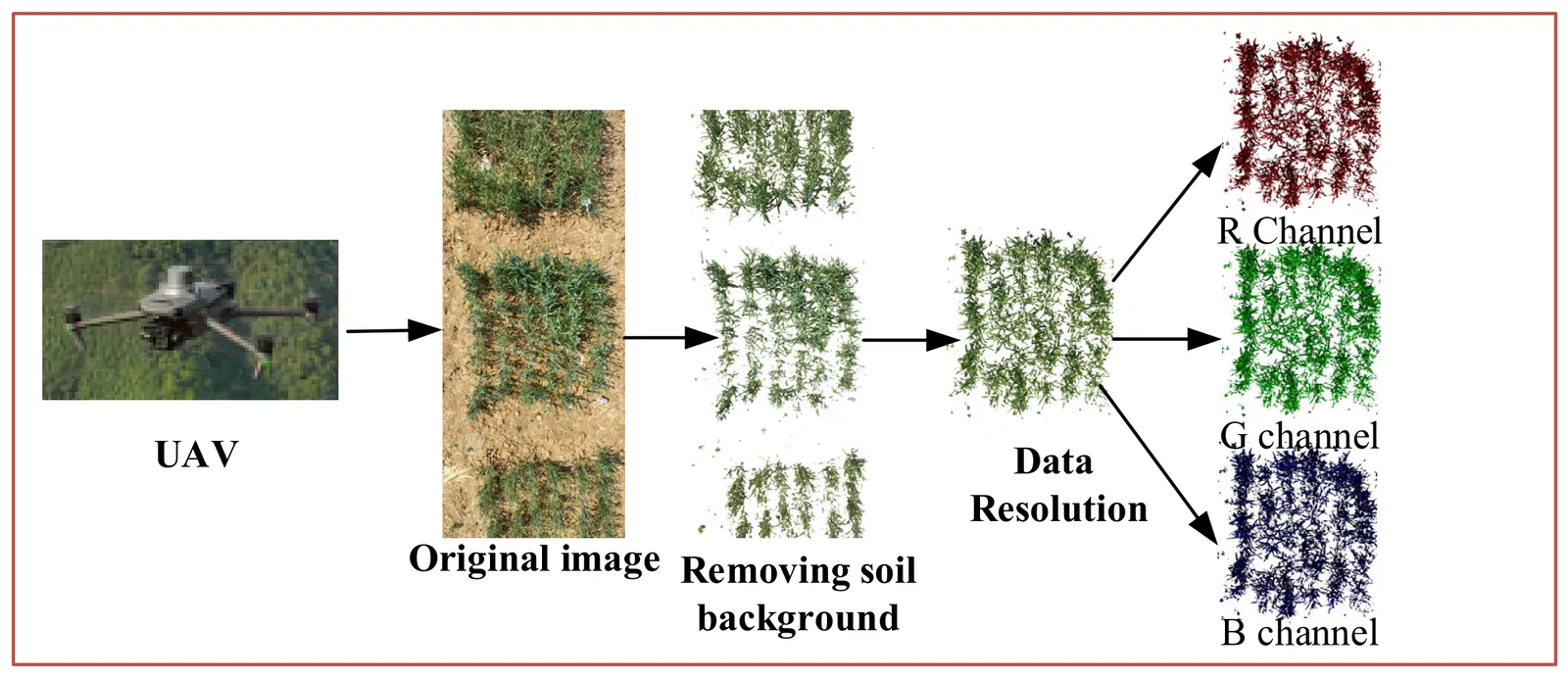
Extraction workflow for the R, G, and B channels of wheat canopy images.

In this study, the acquired wheat canopy images were cropped using Adobe Photoshop to align them with the experimental plots. The processed images were then saved in JPG format. The data preprocessing workflow is shown in Figure 2. To extract the canopy, the G and R channels were extracted from each cropped image to compute the green–red index (GRI), which enhanced the grayscale contrast between vegetation and background. The GRI was calculated as shown in Equation (1). The GRI image was then normalized to a grayscale range of 0–255, and Otsu’s adaptive thresholding algorithm was used to automatically determine the segmentation threshold between vegetation and non-vegetation regions [16]. Finally, a binary mask was generated to extract the vegetation canopy region for subsequent feature extraction.

GRI = (G − R)/(G + R)(1)

A white-panel image was captured for each acquisition batch. For each RGB channel, the mean value of canopy pixels was divided by the corresponding mean value of a shadow-free reference region without overexposure or channel clipping on the paired panel and multiplied by the nominal reflectance of the white reference panel (0.99). For each plot and sampling date, the 14 normalized observations were averaged separately for R, G and B to obtain the daily reference.

(2) $$\rho_{j}=0.99\times \frac{{\overset{-}{I}}_{j}^{\mathrm{canopy}}}{{\overset{-}{I}}_{j}^{\mathrm{white}}},\;j\in \{R,G,B\}$$

where ρ_j_ represents the normalized canopy reflectance in channel j; ${\overset{-}{I}}_{j}^{\mathrm{canopy}}$ is the mean pixel value of the canopy region in channel j; ${\overset{-}{I}}_{j}^{\mathrm{white}}$ is the mean pixel value of a shadow-free reference region without overexposure or channel clipping on the paired white reference panel in channel j; 0.99 is the nominal reflectance of the white reference panel; and j ∈ {R,G,B} denotes the red, green, and blue channels, respectively.

### 2.3. Extraction of Digital Image Parameters from Wheat Canopies

After soil background removal, corresponding feature parameters were extracted from the R, G, and B channel images of the wheat canopy. These features included RGB colour parameters, HSV colour parameters, Lab colour parameters, and BDI-derived shadow parameters, as listed in Table 2. Among these features, RGB-derived statistical parameters can describe canopy leaf-colour intensity, the directional bias of the leaf-colour distribution, and the degree of distributional concentration. HSV-derived colour parameters are relatively insensitive to geometric transformations and remain robust under varying illumination conditions. In canopy image analysis, the Lab colour space separates the lightness component (L) and further characterizes canopy colour variation through the red–green channel (a) and yellow–blue channel (b). BDI provides an ordered representation of RGB-channel differences within each canopy image.

#### 2.3.1. Extraction of Skewed Parameters from the RGB Colour Model

Following the method described by Chen et al. [17], leaf-colour distribution feature parameters were extracted from wheat canopy RGB images using scripts developed in Visual Studio Code (v1.141.0). For the red (R), green (G), blue (B), and grayscale (Y) channels, the mean, median, mode, skewness, and kurtosis were calculated separately, yielding a total of 20 skewed parameters (SP). Among these parameters, the mean reflects the overall colour intensity of the canopy region in the corresponding channel; the median reduces the influence of extreme pixels on feature representation; and the mode describes the dominant colour level in the canopy image. Skewness characterizes the asymmetry of the pixel-value distribution and can indicate whether the colour distribution shifts towards brighter or darker regions. Kurtosis describes the concentration of the pixel-value distribution and the presence of extreme values, and can be used to characterize the uniformity and dispersion of canopy colour distribution. Together, these parameters comprehensively characterize wheat canopy leaf-colour features in terms of colour intensity, dominant colour level, and distributional morphology. To reduce the effects of illumination variation and differences in camera response on colour-intensity features, absolute-value parameters, including the mean, median, and mode of each channel, were normalized by dividing them by the corresponding white reference response values. This normalization improved the comparability of features across different acquisition times and samples.

#### 2.3.2. Extraction of HSV Parameters

Following previous studies on UAV-based RGB feature extraction for crop phenotyping [13], the canopy images were converted from the RGB colour model to the HSV colour model. Hue (H), saturation (S), and value (V) were then calculated, and the mean values of the H, S, and V channels were obtained as HSV colour features for model input, denoted as H_mean_, S_mean_, and V_mean_, respectively. The same colour-space conversion and statistical feature extraction procedures were applied to all samples to ensure feature consistency and comparability across samples.

#### 2.3.3. Extraction of Lab Parameters

To further characterize the colour information of canopy images, the RGB canopy images were converted into the CIE Lab colour space using scripts developed in Visual Studio Code, and statistical features were extracted from the Lab channels. The CIE Lab colour space is an approximately perceptually uniform colour space consisting of the lightness component L, the red–green opponent component a, and the yellow–blue opponent component b. In this colour space, L represents image lightness, a characterizes colour variation along the green–red axis, and b characterizes colour variation along the blue–yellow axis. During feature extraction, the images were first preprocessed using a unified procedure, and the target canopy region was identified. The RGB pixels within the canopy region were then converted into the CIE Lab colour space. Finally, the mean values of the L, a, and b channels were calculated and used as colour features for model input, denoted as Lmean, amean, and bmean, respectively. The same colour-space conversion and statistical feature extraction procedures were applied to all samples to ensure feature consistency and comparability across samples.

#### 2.3.4. Extraction of BDI Parameters

**Figure 3:**
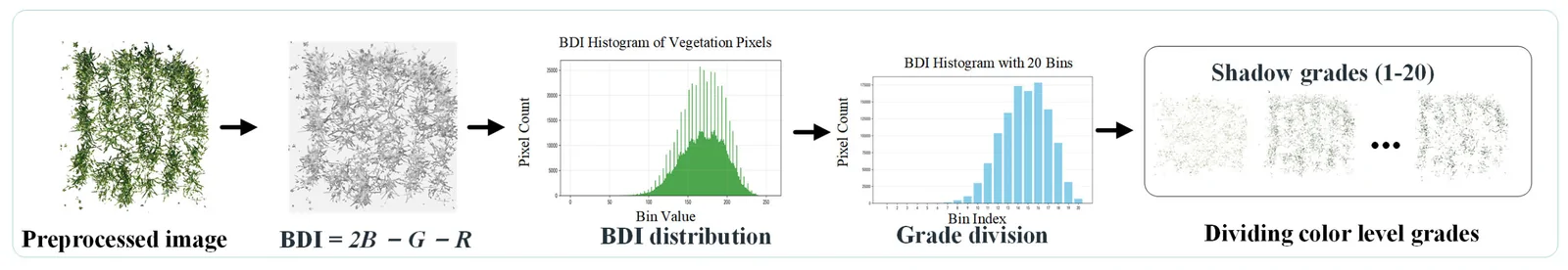
Generation of the wheat canopy BDI map and shadow-grade partitioning.

Following Sharma et al. [15], BDI was used to arrange canopy pixels into ordered grades based on their RGB values. BDI = 2B − G − R captures differences between the blue channel and the red and green channels. The BDI was calculated according to Equation (3), and an example of the resulting BDI map is shown in Figure 3. For each image, canopy BDI values were normalized to 0–255 and divided into 20 ordered grades. Based on the pixel locations associated with each grade, the corresponding regions were mapped back to the original wheat canopy image, enabling the reconstruction of canopy subsets representing different BDI grades. The grades describe variation in BDI within each image.

Two strategies were adopted for modelling with BDI-derived features. In the first strategy, only the minimum, mean, and maximum values of the entire wheat canopy BDI image were used as model inputs (Table 3, Canopy_BDI). These features were denoted as BDImin, BDImean, and BDImax, respectively. In the second strategy, original BDI grades 1–3 and 18–20 were omitted from the graded-feature input because they lay at the ends of the within-image BDI distribution. SP, HSV and Lab features were extracted separately from the retained original grades 4–17, which were renumbered as input grades 1–14. These features formed a 14 × 26 matrix; input grades 10–14 corresponded to original BDI grades 13–17. To investigate the effect of preserving BDI-grade structure, two feature-input formats were considered: (i) a flattened vector obtained by concatenating all features (Fullgrade_flat) and (ii) a matrix-structured representation that retained the original BDI-grade arrangement (Fullgrade_noflat), as summarized in Table 3.

BDI = 2B − G − R(3)

### 2.4. Construction of Reflectance Correction Models

The four categories of features listed in Table 2 served as predictor variables, with the R, G, and B reflectance values as the corresponding responses. To evaluate the effects of different feature representations on the prediction of R-, G-, and B-channel reflectance, four feature-input configurations were constructed: (i) a 26-dimensional whole-canopy feature vector (Canopy); (ii) a 29-dimensional whole-canopy feature vector incorporating BDI-derived features (Canopy_BDI); (iii) a 364-dimensional flattened feature vector obtained from shadow-grade canopy features (Fullgrade_flat); and (iv) a structured 14 × 26 feature matrix that preserved the original shadow-grade feature arrangement (Fullgrade_noflat). The first two feature-input configurations were used to evaluate the predictive capability of leaf-colour distribution features and the BDI-derived shadow descriptor. The third configuration assessed the effect of directly concatenating shadow-grade features into a high-dimensional vector. The fourth configuration was designed to investigate whether preserving the structural ordering of shadow-grade features could improve model generalization by retaining information on the continuous variation of canopy illumination conditions. The feature-input configurations are summarized in Table 3.

Five machine learning methods were employed to construct reflectance correction models in this study: a multilayer perceptron (MLP), a Kolmogorov–Arnold network (KAN), a back-propagation neural network (BPNN), a long short-term memory network (LSTM), and a one-dimensional convolutional neural network (1D-CNN). The MLP served as a baseline multilayer feedforward network, as its capability for nonlinear function approximation has been well established in previous studies [18,19]. The BPNN was employed to evaluate the nonlinear fitting capability for image statistical features and was trained using an error back-propagation mechanism [20,21]. The KAN was employed to model complex nonlinear feature–response relationships using a Kolmogorov–Arnold network architecture with parameterized edge functions [22,23]. The LSTM was employed to model dependencies within ordered feature sequences [24,25], and the 1D-CNN extracts local patterns from ordered feature sequences through one-dimensional convolution, making it well suited for structured inputs based on canopy shadow grades [26]. All models were trained and evaluated using the same data-splitting strategy and evaluation metrics, with model selection based on validation loss to ensure fair comparison.

The MLP consisted of multiple fully connected layers with ReLU activations and dropout, and simultaneously predicted the R-, G-, and B-channel reflectance values through a three-dimensional linear output layer. For the Canopy and Canopy_BDI input configurations, the hidden-layer architecture was set to [128, 64, 32]; for Fullgrade_flat, it was expanded to [256, 128, 64] to accommodate the increased input dimensionality. The BPNN, designed as a shallow baseline with a single hidden layer and ReLU activations, followed established practices for crop trait estimation. Its hidden units were searched within 10–30 for Canopy/Canopy_BDI and 60–150 for Fullgrade_flat. The KAN, which defines learnable nonlinear functions on network edges parameterized by B-splines, was configured with a grid size of 5, a spline order of 3, and SiLU as the base activation. Its hidden-layer size was tuned within 10–30 for Canopy/Canopy_BDI and 20–50 for Fullgrade_flat. The LSTM employed a two-layer unidirectional architecture with 64 hidden units per layer and a dropout rate of 0.1; the hidden state from the last time step was passed through layer normalization, dropout, and a linear output layer. The 1D-CNN consisted of two convolutional blocks (64 and 128 channels, kernel size 3), each followed by batch normalization and ReLU, with global average pooling before the final linear layer.

For the Fullgrade_noflat configuration, the input was organized as a 14 × 26 structured feature matrix, where 14 corresponds to the retained BDI-based shadow grades and 26 represents the colour and statistical features within each grade, preserving the inherent ordering information. The MLP, BPNN, and KAN adopted a hierarchical “within-grade encoding–across-grade aggregation” paradigm: the 26-dimensional feature vectors within each grade were nonlinearly encoded, followed by attention-based pooling across grades. Their hyperparameters were configured as MLP (hidden units = 128, embedding dimension = 64), BPNN (hidden units = 96), and KAN (hidden units = 48, embedding dimension = 32, grid size = 5, spline order = 3). The LSTM and 1D-CNN treated the 14 grades as an ordered sequence of increasing BDI values. The LSTM used the same two-layer unidirectional architecture with 64 hidden units per layer and dropout 0.1, while the 1D-CNN used the same convolutional configuration ([64, 128] channels, kernel size 3) with global average pooling along the grade dimension. All models ultimately output corrected R, G, and B reflectance values.

To ensure fair comparison, the training strategy was kept consistent across input configurations. For the MLP, LSTM, and 1D-CNN models, the AdamW optimizer was employed with an initial learning rate of 0.005, a weight decay of 1 × 10^−4^, and a batch size of 64. The BPNN and KAN models were optimized using the Adam optimizer. For the BPNN, the learning rate was set to 0.005 for the Canopy, Canopy_BDI, and Fullgrade_flat configurations, and adjusted to 0.01 for the Fullgrade_noflat configuration. The KAN model was trained with a learning rate of 0.01 and a batch size of 32. All models employed early stopping based on validation loss, with a patience of 20 epochs and a maximum training budget of 150–300 epochs. For the LSTM and 1D-CNN models, a ReduceLROnPlateau scheduler was applied to adjust the learning rate dynamically, with a patience of 5 and a decay factor of 0.5. Gradient clipping with a threshold of 5.0 was also used to enhance training stability. Each model predicted three plot-level daily-reference values (R, G and B) from pixel-derived features of one image.

To prevent data leakage, a group-based splitting strategy was adopted based on field plots, ensuring that samples from the same plot were assigned to a single subset (training, validation, or test). A 5-fold group-based cross-validation was employed: five mutually exclusive subsets were constructed by plot_id; in each fold, one subset served as the test set and the remaining as training–validation, from which a validation set was further partitioned for early stopping, learning rate scheduling, and model selection. The model was evaluated on the held-out test set of each fold. This strategy prevents samples from the same plot from appearing in both training and test sets, providing a realistic evaluation of generalization to unseen plots, with the test set strictly excluded from all preprocessing and tuning procedures [27].

### 2.5. Evaluation Metrics and Feature Contribution Analysis

#### 2.5.1. Evaluation Metrics for Correction Models

To comprehensively assess the predictive performance of the model in estimating R-, G-, and B-channel reflectance, three widely used metrics, namely root mean square error (RMSE), mean absolute error (MAE), and coefficient of determination (R^2^), were employed, with their mathematical definitions provided in Equations (4)–(6). Specifically, RMSE and MAE quantify the magnitude of prediction errors, with lower values indicating improved predictive performance. R^2^ measures the proportion of variance in the observed data explained by the model, with values closer to 1 indicating a better model fit.

(4) $$\begin{array}{c} \mathrm{RMSE}=\sqrt{\frac{1}{3n}{\sum_{i=1}^{n}\sum_{j\in \{R,G,B\}}\left(y_{\mathrm{ij}}-{\hat{y}}_{\mathrm{ij}}\right)}^{2}} \end{array}$$

(5) $$\begin{array}{c} \mathrm{MAE}=\frac{1}{3n}\sum_{i=1}^{n}\sum_{j\in \{R,G,B\}}\left|y_{\mathrm{ij}}-{\hat{y}}_{\mathrm{ij}}\right| \end{array}$$

(6) $$\begin{array}{c} R^{2}=1-\frac{{\sum_{i=1}^{n}\left(y_{\mathrm{ij}}-{\hat{y}}_{\mathrm{ij}}\right)}^{2}}{{\sum_{i=1}^{n}\left(y_{\mathrm{ij}}-{\overset{-}{y}}_{j}\right)}^{2}},\;j\in \{R,G,B\} \end{array}$$

Here, *y_ij_* denotes the daily reference value of the *i*-th sample in the *j*-th channel, while ${\hat{y}}_{\mathrm{ij}}$ represents the corresponding predicted value. *n* is the number of samples, *j* ∈ {*R*,*G*,*B*} denotes the RGB channel index. ${\bar{y}}_{j}$ is the mean of the reference values across the *n* samples in channel *j*.

The R^2^ values for the R-, G-, and B-channels are denoted as *R*^2^_*R*_, *R*^2^_*G*_, and *R*^2^_*B*_, respectively, and the average R^2^ across the three channels is denoted as $R_{\mathrm{ave}}^{2}$, as given in Equation (7).

(7) $$\begin{array}{c} R_{\mathrm{ave}}^{2}=\frac{1}{3}\left(R_{R}^{2}+R_{G}^{2}+R_{B}^{2}\right) \end{array}$$

#### 2.5.2. Feature Contribution Analysis

In this study, the SHapley Additive exPlanations (SHAP) method was employed to conduct feature contribution analysis. SHAP is grounded in Shapley values from cooperative game theory, where each input feature is regarded as a “player” contributing to the prediction. It quantifies the marginal contribution of each feature across all possible subsets of features and assigns a corresponding importance value to each variable [28]. Larger absolute SHAP values indicate a stronger influence of the corresponding feature on model predictions, while the sign indicates whether the feature increases or decreases the predicted value. Since the heatmaps in this study are based on absolute SHAP values, they are primarily used to compare feature contribution strength rather than to determine the direction of the effect.

For models that preserve the shadow-grade structure, the input is a three-dimensional tensor of size (N × 14 × 26), where N denotes the number of samples, 14 represents the number of ordered shadow-grade bins, and 26 corresponds to the number of representative features extracted within each bin. The model outputs predicted reflectance values for the R-, G-, and B-channels. In this study, SHAP values for each bin–feature combination in the test samples were computed using GradientExplainer, with background samples derived from the training set. To assess the relative importance of different variables, absolute SHAP values were aggregated along two dimensions. Specifically, averaging across the sample and bin dimensions produces the overall contribution of the 26 input features, while averaging across the sample and feature dimensions yields the overall contribution of the 14 shadow-grade bins. The heatmaps visualize mean absolute SHAP values for the R-, G-, and B-channels, with “Overall” indicating the channel-wise averaged contribution. Larger values correspond to a stronger average impact of the corresponding feature or shadow-grade bin on model predictions.

## 3. Results

### 3.1. Performance of Wheat Canopy Reflectance Correction Models

To systematically assess the impact of different feature input strategies on regression performance, four input configurations (Canopy, Canopy_BDI, Fullgrade_flat, and Fullgrade_noflat) were evaluated using five models (MLP, KAN, BPNN, LSTM, and 1D-CNN). Model performance was assessed on the test set using the coefficient of determination (test R^2^), root mean square error (test RMSE), and mean absolute error (test MAE).

**Figure 4:**
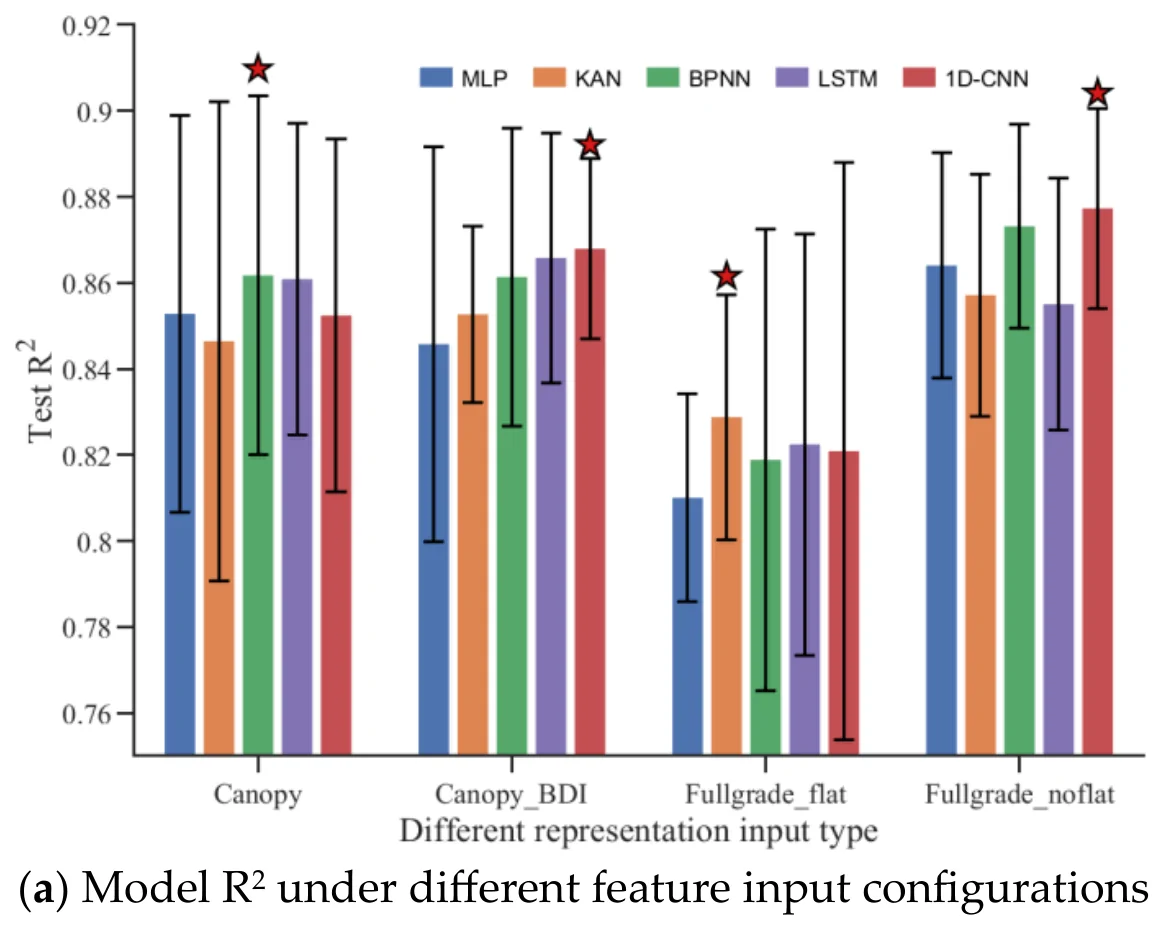

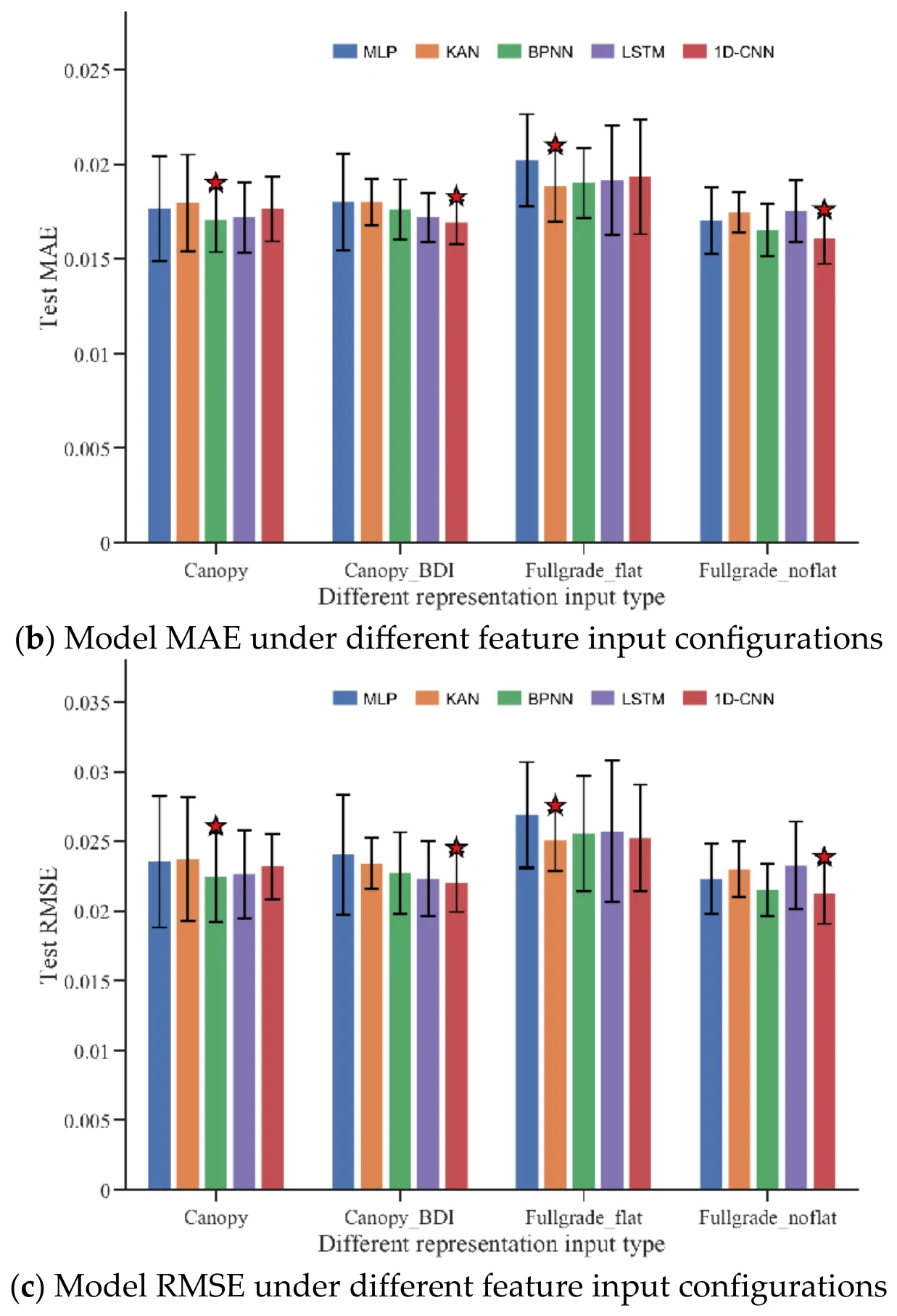
Model prediction performance under different feature input configurations: (**a**) R^2^, (**b**) MAE, and (**c**) RMSE. Bars and error bars denote mean values and standard deviations, respectively. Red stars highlight the best-performing model under each input configuration for the corresponding metric. Higher R^2^ and lower RMSE/MAE indicate superior performance.

The model prediction performance under different feature input configurations is illustrated in Figure 4, whereas Table 4 summarizes the best-performing models for each configuration. A comprehensive comparison of model performance indicates that the Fullgrade_noflat input combined with the 1D-CNN model achieved the highest test R^2^ (0.8773) and the lowest errors (test RMSE = 0.0213, test MAE = 0.0161), outperforming Canopy (best test R^2^ = 0.8617) and Canopy_BDI (best test R^2^ = 0.8679). The experimental results demonstrate that preserving the feature structure substantially improves model performance. Fullgrade_noflat, which retains the original 14-row structure, achieved a higher average test R^2^ of 0.8773, significantly outperforming Fullgrade_flat, where the features were flattened into a 364-dimensional vector (average test R^2^ = 0.8202). These results suggest that increasing feature dimensionality without preserving structural information may lead to degraded predictive performance, highlighting that a larger number of features does not necessarily imply richer or more informative representations. Furthermore, the optimal model differs across input configurations. In Canopy and Fullgrade_flat, the optimal models selected based on validation loss and test R^2^ were inconsistent, whereas Fullgrade_noflat consistently selected 1D-CNN as the optimal model under both criteria, indicating improved generalization consistency and better compatibility between model architecture and input structure.

In terms of leaf colour skewness features and the BDI index, Canopy_BDI yielded a modest but consistent improvement over Canopy, with an average cross-model test R^2^ increase of approximately +0.0039 (from 0.8617 to 0.8656). The best-performing configuration improved from 0.8617 (BPNN) to 0.8679 (1D-CNN). Although the magnitude of improvement was limited, the consistently positive trend across models suggests a stable contribution of BDI-related features.

### 3.2. Feature Contribution Analysis of the Best Correction Model

**Figure 5:**
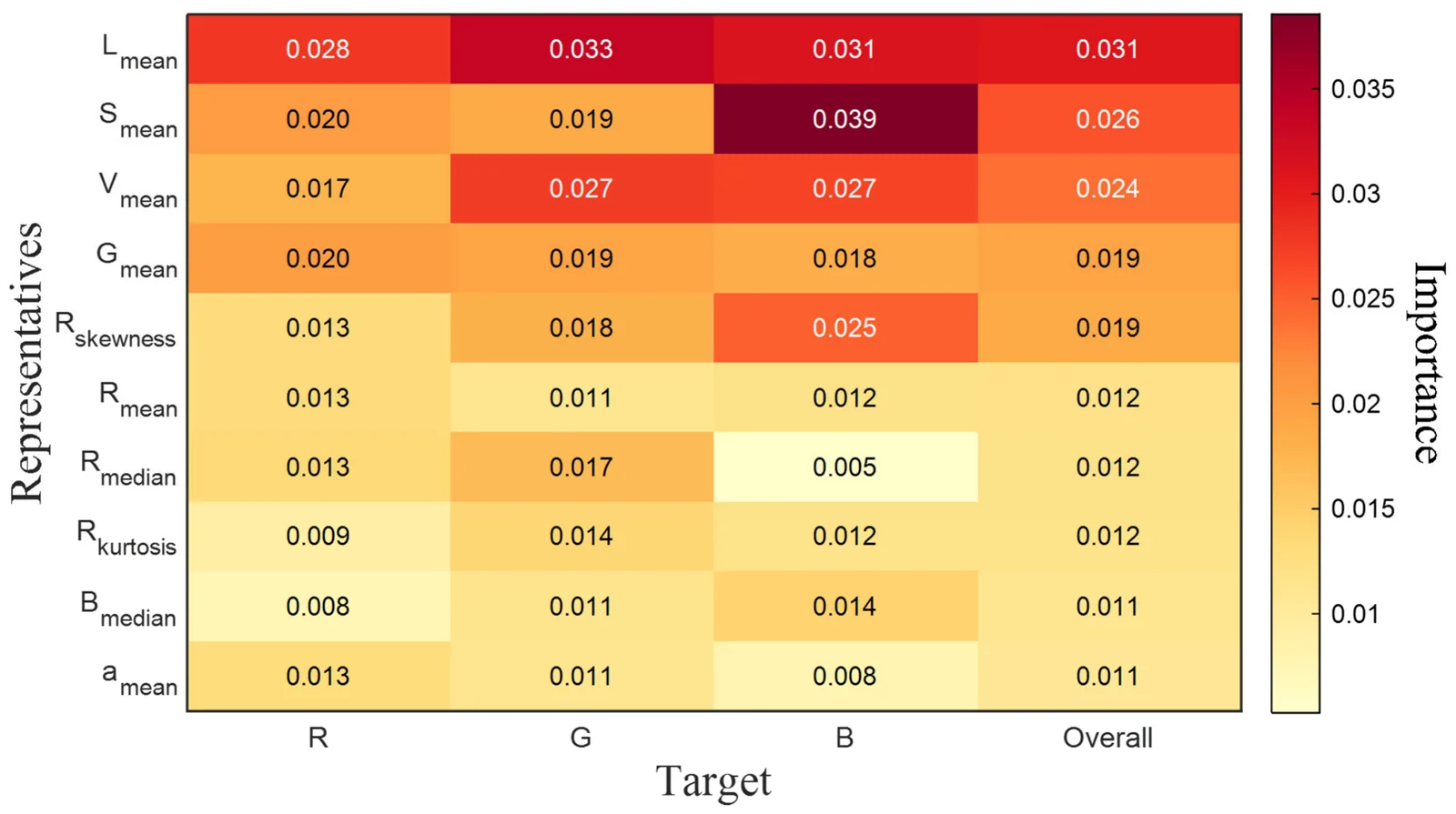
Feature importance analysis based on SHAP values.

Feature contribution analysis was performed on the best correction model, defined as the combination of Fullgrade_noflat and the 1D-CNN model (hereafter referred to as the best correction model). The SHAP feature importance heatmap is presented in Figure 5. L_mean_, S_mean_ and V_mean_ showed relatively high mean absolute SHAP values in the fitted model. In particular, L_mean_ consistently contributes strongly across R-, G-, and B-channel predictions as well as the averaged performance, indicating that brightness-related information contributed to predictions in this dataset. V_mean_ exhibits a similar pattern and is highly consistent with L_mean_, suggesting that brightness-related information remained relevant when the 14 ordered grades were included. In contrast, S_mean_ exhibits a notably stronger contribution to B-channel predictions, suggesting that saturation-related variations are more influential in specific channel estimations.

The feature importance analysis indicates that colour space components such as L_mean_, V_mean_, and S_mean_ consistently contribute strongly across multiple prediction targets, indicating that the fitted model used brightness- and saturation-related features in predicting the daily reference. Specifically, L_mean_ encodes image brightness information, whereas V_mean_ and S_mean_ represent variations in luminance and saturation, respectively. The relatively high importance of these features suggests that, during reflectance prediction, the model not only relies on raw RGB channel intensities but also effectively integrates complementary information from multiple colour spaces that capture canopy brightness variations and colour-related properties.

Meanwhile, RGB statistical features derived from whiteboard normalization, including G_mean_, R_mean_, and R_median_, also show high feature importance, indicating that relative channel intensities continue to serve as fundamental predictors in RGB reflectance estimation. The contributions of higher-order statistical features such as R_skewness_ and R_kurtosis_ further indicate that the model not only exploits central tendency measures such as mean and median, but also integrates distributional shape information of pixel values. Specifically, skewness and kurtosis characterize the asymmetry and peakedness of the colour distribution, respectively. These findings indicate that the heterogeneity of canopy colour distributions plays a key role in influencing reflectance estimation accuracy.

**Figure 6:**
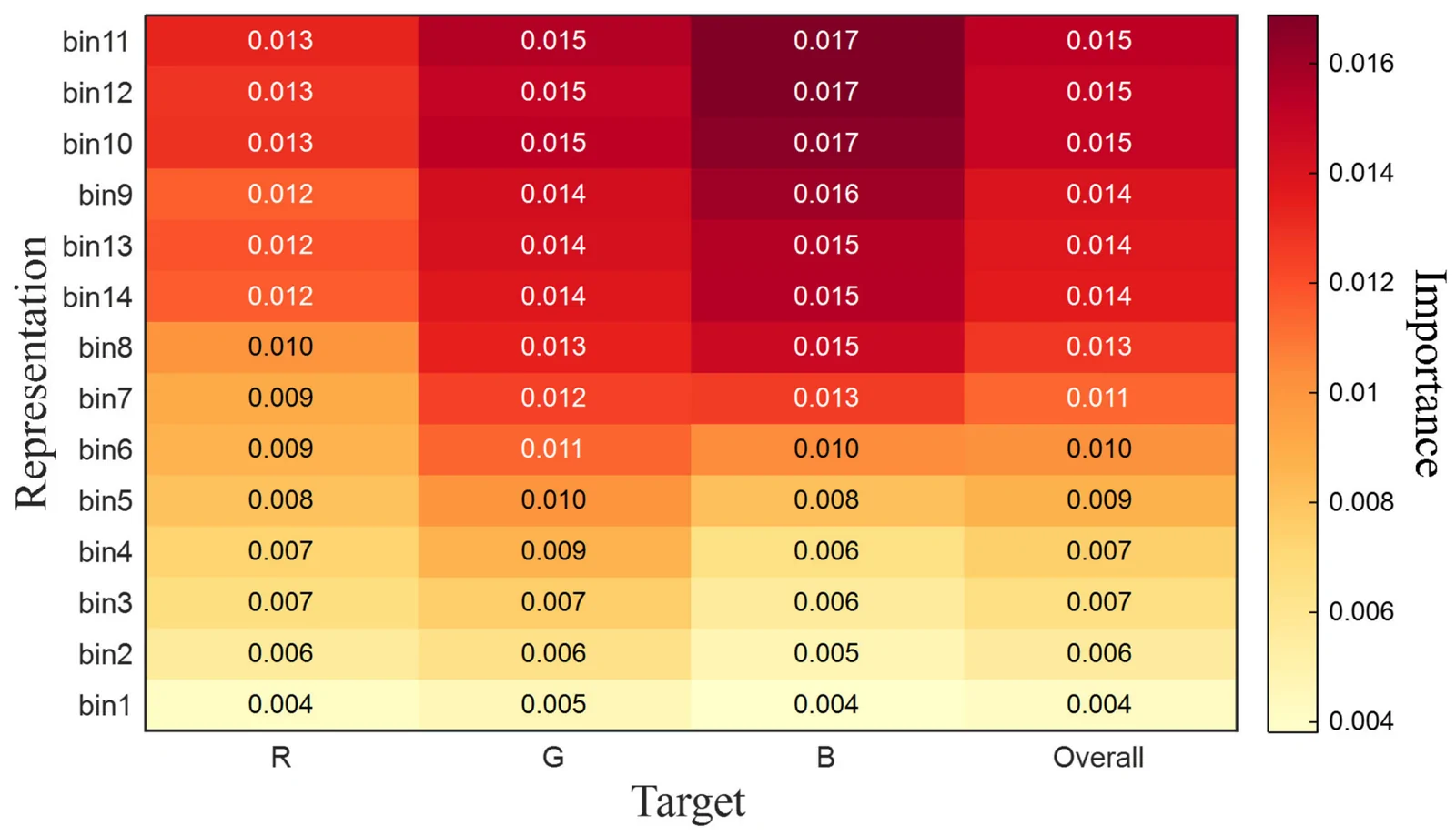
Significance analysis of canopy data across different shadow grades.

The significance analysis of canopy data across different shadow grades is presented in Figure 6. The bin importance results indicate that the correction model exhibits non-uniform utilization of canopy information across different BDI levels. High-importance regions are primarily concentrated within bins 10–14, among which bins 10, 11, and 12 show the most consistent and stable contributions across samples. This suggests that the mid-to-high and high BDI response ranges contain more discriminative information and make a greater contribution to the target prediction outputs.

Furthermore, this result supports the rationale for preserving the 14-row BDI sequence input from the perspective of model architecture. Owing to the inherent ordinal structure of the 14 bins arranged from low to high BDI responses, the 1D-CNN can effectively capture local continuity patterns between adjacent bins, including brightness gradients, saturation variations, and gradual transitions in RGB leaf colour distribution properties. In contrast, flattened inputs decouple bin indices and feature types into independent variables, thereby weakening the ordinal structure of BDI levels and encouraging the model to rely more heavily on fragmented, bin-specific feature cues. Therefore, structured inputs better preserve the continuous colour stratification induced by BDI-based layering, enabling the 1D-CNN to more effectively learn local inter-bin patterns and hierarchical relationships.

### 3.3. Correction Results of the Best Model

**Figure 7:**
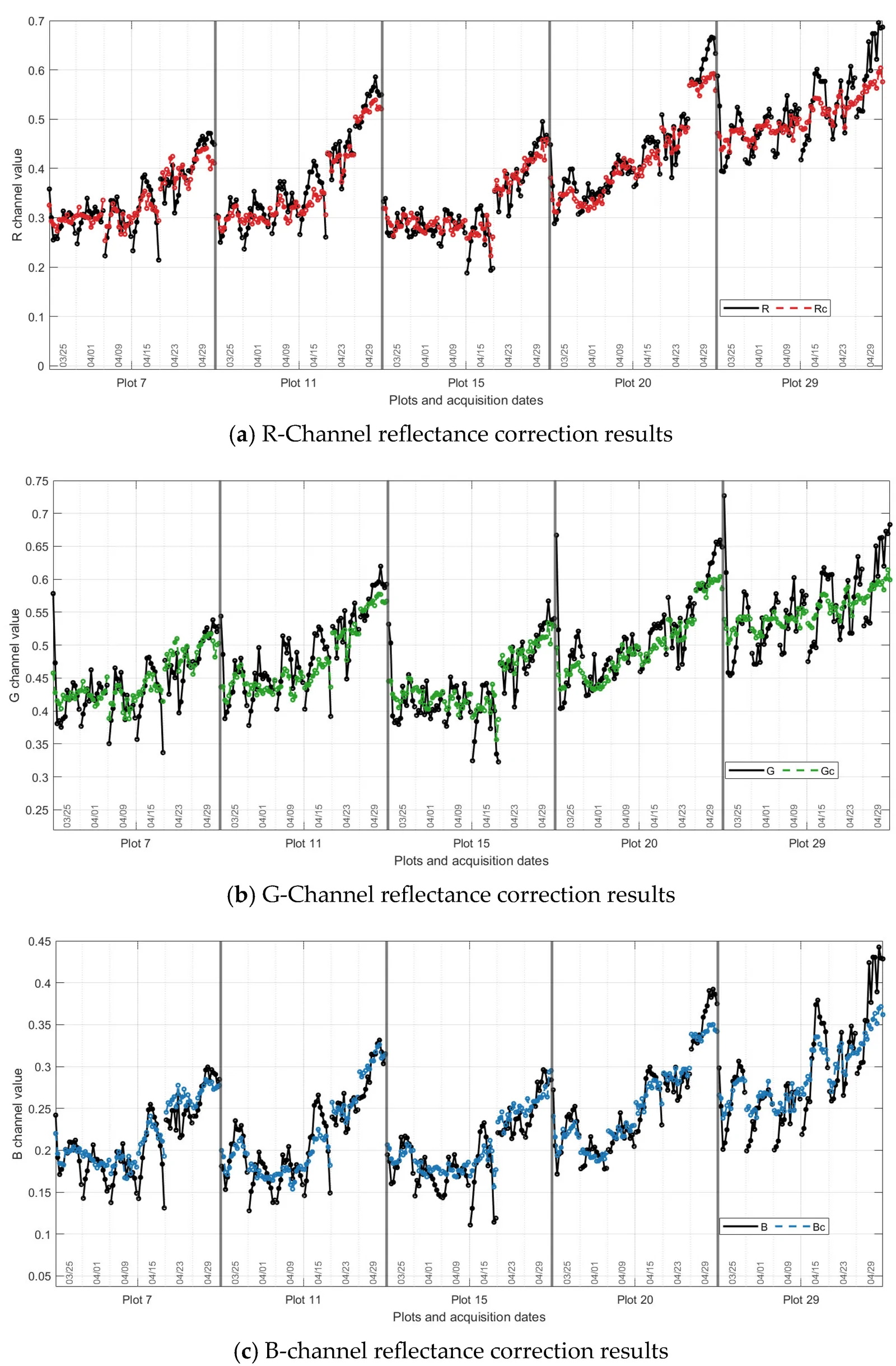
Reflectance correction results for the R-, G-, and B-channels. (**a**) R-channel reflectance correction results; (**b**) G-channel reflectance correction results; (**c**) B-channel reflectance correction results. Note: Rc, Gc and Bc denote the predicted (corrected) reflectance of the R, G and B channels, respectively, while R, G and B denote the original (uncorrected) reflectance of the corresponding channels.

**Figure 8:**
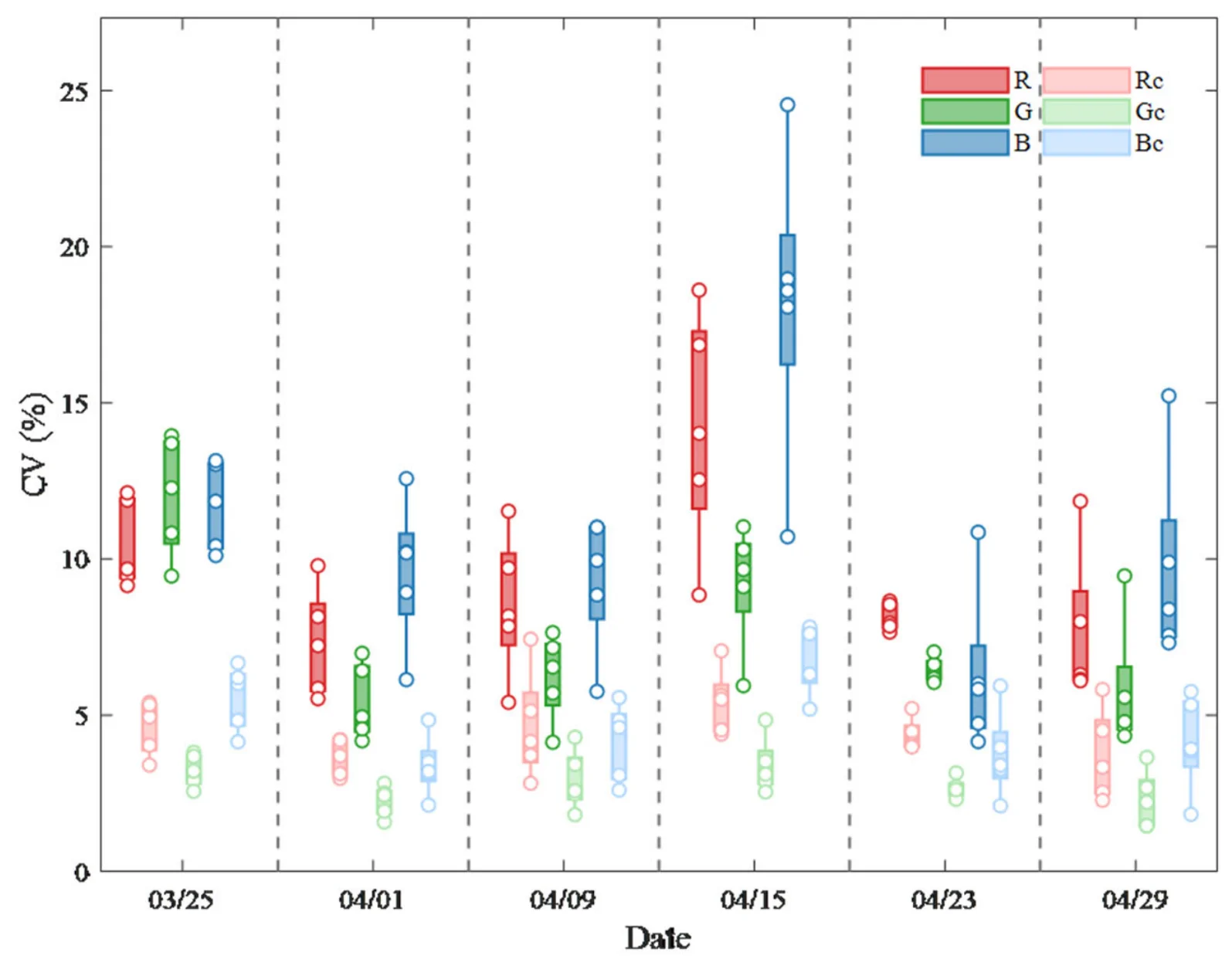
Coefficient of variation of reflectance before and after correction across different observation dates. Note: Rc, Gc and Bc denote the predicted (corrected) reflectance of the R, G and B channels, respectively, while R, G and B denote the original (uncorrected) reflectance of the corresponding channels.

The reflectance correction results for the R-, G-, and B-channels produced by the best correction model are presented in Figure 7. Here, R_c_, G_c_ and B_c_ denote the predicted values of the R, G and B channels, respectively. Compared with the original RGB channel series, the predicted outputs show a significant reduction in the coefficient of variation across all three channels. Specifically, the coefficient of variation (CV) of reflectance measurements for the B-, G-, and R-channels decreased from 10.81%, 7.52%, and 9.39% to 4.65%, 2.79%, and 4.40%, respectively, corresponding to reductions of 56.98%, 62.85%, and 53.09%. The CV of reflectance before and after correction across different observation dates is shown in Figure 8.

Compared with the original observations, the model-predicted RGB values showed smaller within-day fluctuations in the example plots (Figure 7). For Plots 20 and 29, the main numerical changes across sampling dates remained visible.

The correction performance of the best model differs across the R, G, and B channels, as shown in Table 5. The average R^2^ of Rc is the highest (0.9024), followed by Bc (0.8736), while Gc shows the lowest value (0.8008). Meanwhile, Gc exhibits the largest standard deviation of R^2^, indicating relatively lower prediction stability for the green channel across different models and folds.

### 3.4. Comparison with Other Correction Methods

This study compares shadow compensation correction methods based on wheat canopy BDI, grayscale, and SDI images, referred to as BDI-Otsu, RGB-Y-Otsu, and SDI local, respectively. BDI-Otsu constructs a shadow-sensitive index based on inter-channel differences in RGB space and employs Otsu’s automatic thresholding method to segment shadow regions [15,16]. RGB-Y-Otsu identifies shadow regions with low luminance using a luminance component derived from RGB channels, where the computation follows the classical RGB-to-luminance conversion model defined by ITU-R [29]. SDI local adopts the RGB-based SDI shadow detection method proposed by Liu et al. [11], where SDI = ω|2G − B − R| + εG is used to enhance shadow-related features, followed by threshold-based segmentation and local window compensation. Overall, these three methods represent three types of shadow correction strategies, namely colour-difference indices, brightness-based thresholding, and local adaptive compensation, and serve as benchmark baselines for evaluating the effectiveness of data-driven methods.

To ensure a fair comparison, all methods were evaluated on the same test set, and their performance was summarized across the R, G, and B channels. The evaluation metrics include RMSE, MAE, and CV, where RMSE and MAE quantify correction errors, and CV reflects within-group stability. All results are reported as mean ± standard deviation in Table 6. In this dataset, the proposed model had the lowest RMSE and MAE among the evaluated implementations and reduced within-day CV within plot–date groups. The comparison in Table 6 is specific to this prediction target and the three RGB shadow-compensation implementations tested here.

## 4. Discussion

### 4.1. Comparative Analysis of Models and Feature Input Configurations

This study addresses the issue of RGB reflectance fluctuations caused by the interaction between wheat canopy structure and solar illumination, and develops a canopy image reflectance correction model based on leaf colour–shadow structural features. Due to the combined effects of solar elevation angle, canopy occlusion, leaf orientation, and local illumination gradients, raw RGB reflectance in field images not only contains intrinsic optical information of the canopy but also incorporates non-target variations induced by shadows and viewing geometry. The model was therefore trained to predict a separate daily reference for each plot and sampling date. The reduced within-day CV indicates greater consistency of repeated RGB observations; independent trait measurements are needed to assess how well agronomic differences are retained. Fivefold cross-validation grouped by plot ID kept all observations of a plot in the same fold, allowing performance to be evaluated on plots not used for training within the present site and season.

From the results across different feature input configurations, the Canopy input achieves strong predictive performance, with a best Test R^2^ of 0.8617, suggesting that canopy-level statistical descriptors such as SP, HSV, and Lab effectively capture the nonlinear relationship between RGB reflectance and canopy colour distribution. After incorporating global BDI statistics, the Canopy_BDI configuration improves the best Test R^2^ to 0.8679, while the cross-model mean Test R^2^ increases from 0.8617 to 0.8656, suggesting that BDI provides complementary information for characterizing shadow and illumination variations in canopy imagery. However, the improvement remains limited, indicating that the three global statistics (BDI_min_, BDI_mean_, and BDI_max_) summarize the overall BDI distribution but do not retain the colour statistics within individual BDI grades.

Notably, although Fullgrade_flat increases the feature dimensionality to 14 × 26, its best Test R^2^ reaches only 0.8288, which is inferior to both Canopy and Canopy_BDI, highlighting the limited benefit of simply increasing feature dimensionality without preserving structural information. This indicates that, under the current sample size and model configuration, simply increasing the number of features does not necessarily lead to improved model performance. When the features across 14 shadow levels are directly flattened, the inherent ordinal relationship among BDI levels is weakened, and the model treats them as high-dimensional independent variables for fitting, which may introduce redundant information and noise and reduce generalization stability. Therefore, the results of Fullgrade_flat indicate that the value of shadow-level features does not lie merely in the increased number of features, but also depends on whether the model can effectively exploit their structural relationships. In contrast, the combination of Fullgrade_noflat and 1D-CNN achieves the best performance, with a Test R^2^ of 0.8773, a Test RMSE of 0.0213, and a Test MAE of 0.0161, representing the optimal configuration among the four input strategies. This result indicates that retaining the structured input form of 14 BDI shadow levels is more conducive to enabling the model to learn relationships among features from adjacent BDI grades. 1D-CNN treats the 14 BDI bins as a one-dimensional sequence, enabling the extraction of local variation patterns across adjacent shadow levels; consequently, its inductive bias aligns more closely with the inherent structure of Fullgrade_noflat. Compared with the flattened Fullgrade_flat input, this structured modelling approach not only preserves the ordinal relationships among shadow levels but also reduces the model’s reliance on isolated high-dimensional variables, thereby improving prediction stability and generalization capability.

SHAP analysis was used to examine feature contributions in the fitted model. The SHAP results indicate that the most influential features in the optimal correction model include L_mean_, S_mean_, and V_mean_. Among them, L_mean_ consistently shows high contributions across the R, G, and B targets as well as the overall prediction, indicating that luminance-related information contributed to predictions in this dataset. V_mean_ reflects image brightness variations, while S_mean_ captures changes in colour saturation. Together, these features indicate that the model used brightness and saturation information in the present dataset. G_mean_, R_mean_, R_median_, R_skewness_ and R_kurtosis_ also contributed to the predictions. Together, these correlated features describe colour intensity and its distribution in the fitted model.

From the perspective of BDI shadow-level contributions, bins 10–14—particularly bins 10, 11, and 12—show relatively stable importance, indicating that the mid-to-high BDI response regions contain stronger discriminative information. The lower contribution of low BDI regions does not indicate that they are uninformative, but rather suggests that, under the current data distribution and model configuration, their discriminative power for target reflectance variation is relatively limited.

### 4.2. Analysis of Correction Performance Across Different Channels

The correction performance of the optimal model differs across RGB channels, with the results reported in Table 5. Rc achieves the highest mean R^2^ (0.9024), followed by Bc (0.8736), whereas Gc has the lowest mean R^2^ (0.8008). Meanwhile, Gc exhibits the highest standard deviation of R^2^, suggesting lower prediction stability of the green channel across cross-validation folds. The lower mean R^2^ for Gc may reflect several interacting factors, including leaf colour, canopy structure and illumination; their separate contributions were not measured in this study.

The R channel had the highest mean R^2^ in Table 5, although the contribution of chlorophyll absorption and canopy structure to this result was not measured separately. In the R channel, features such as Rskewness, Rmean, and amean showed relatively high SHAP contributions, indicating that the fitted model used both channel intensity and distributional information. The mean R^2^ of Bc is 0.8736, lower than that of Rc but higher than that of Gc. Illumination, camera colour processing and shadowing may contribute to B-channel variation, although their individual effects were not measured here [30,31]. However, in the optimal model, features such as V_mean_, S_mean_, and B_mean_ still exhibit relatively high contributions, indicating that the fitted model used brightness, saturation and B-channel statistics. The BDI-grade representation also contributed to B-channel prediction in this dataset.

From the perspective of the coefficient of variation before and after correction, the optimal model significantly reduces short-term fluctuations across all three channels. The CV values of the B, G, and R channels decrease from 10.81%, 7.52%, and 9.39% to 4.65%, 2.79%, and 4.40%, respectively, corresponding to reductions of 56.98%, 62.85%, and 53.09%. The relative reduction in CV was greatest for the G channel, whereas the B channel retained the highest CV after correction.

### 4.3. Limitations and Future Work

Although the optimal correction model proposed in this study achieves satisfactory reflectance correction performance on the current dataset, several limitations still exist. First, the data used in this study were collected from a single experimental site and one growing season. Although several nitrogen treatments and sampling dates were included, performance in other regions, years, cultivars, sensors and weather conditions requires independent validation. Future work should incorporate multi-site, multi-year, and multi-sensor data and establish an external independent test set to systematically evaluate the model’s cross-domain generalization capability. Second, BDI was used to organize pixel values into ordered grades, but its correspondence to physical shadow states and the effect of omitting the extreme grades remain to be tested. Dataset-wide RGB clipping and the relationships between BDI-grade distributions and LAI or canopy cover were not evaluated. Since canopy reflectance variations are fundamentally driven by both canopy structure and illumination geometry, future work could incorporate solar elevation angle, solar azimuth angle, incident light intensity, acquisition time, and weather conditions as auxiliary inputs to further enhance the model’s interpretability under varying illumination conditions. Third, the comparison with BDI-Otsu, RGB-Y-Otsu and SDI local in Table 6 concerns their RGB reference implementations on the present dataset; physical and radiometric methods were not evaluated under the same target and data split. Finally, although the current optimal model achieves good predictive accuracy and structural interpretability, it remains an empirical data-driven model. SHAP analysis and bin importance results can reveal the features and shadow levels primarily relied upon by the model; however, these interpretations should be regarded as model-level correlational evidence rather than direct causal proof of plant physiological mechanisms. Further work should compare the daily reference with independent reflectance and agronomic measurements to assess its physical accuracy and value for trait analysis [32].

## 5. Conclusions

This study addresses the problem of RGB reflectance variations caused by the interaction between wheat canopy structure and solar radiation under natural illumination conditions, and develops a reflectance correction model guided by leaf-colour and shadow-structure features. Based on a systematic comparison of four feature input strategies (Canopy, Canopy_BDI, Fullgrade_flat, and Fullgrade_noflat) and five machine learning models (MLP, KAN, BPNN, LSTM, and 1D-CNN), the following conclusions can be drawn.

(1) Canopy leaf-colour distribution features can effectively calibrate RGB reflectance, with the best model achieving a Test R^2^ of 0.8617 under the Canopy input. After incorporating global BDI statistics, the Canopy_BDI model improves the best Test R^2^ to 0.8679, indicating that BDI provides complementary information for shadow and illumination variations; however, the performance gain is limited when only BDI_min_, BDI_mean_, and BDI_max_ are used.
(2) Simply increasing feature dimensionality does not guarantee performance improvement. Fullgrade_flat directly flattens 14 shadow levels with 26-dimensional features into a 364-dimensional input; however, its best Test R^2^ is 0.8288, which is lower than that of Canopy and Canopy_BDI. This result indicates that the effectiveness of shadow-level features depends on preserving their structural relationships rather than merely concatenating high-dimensional features.
(3) The Fullgrade_noflat input, which preserves the BDI shadow-level structure, is best aligned with the 1D-CNN model and achieves the highest correction performance. The Fullgrade_noflat–1D-CNN model obtains a Test R^2^ of 0.8773, a Test RMSE of 0.0213, and a Test MAE of 0.0161, indicating that the 1D-CNN can effectively learn local continuous variation patterns between adjacent shadow levels and represents the optimal wheat canopy RGB reflectance correction method in this study.
(4) Feature importance analysis indicates that L_mean_, V_mean_, and S_mean_ are the most important features in the correction model, suggesting that brightness- and saturation-related information is critical for reflectance correction. The contributions of BDI levels are mainly concentrated in bins 10–14, particularly bins 10, 11, and 12, indicating that mid-to-high BDI response regions contain strong discriminative information and showing the relatively high contribution of these grades to predictions in the fitted model.
(5) The optimal model effectively reduces diurnal variability in RGB reflectance. The coefficients of variation (CV) for the B, G, and R channels decrease from 10.81%, 7.52%, and 9.39% to 4.65%, 2.79%, and 4.40%, corresponding to reductions of 56.98%, 62.85%, and 53.09%, respectively.

In this study, the Fullgrade_noflat input combined with 1D-CNN gave the best prediction of the plot-specific daily mean RGB reference among the evaluated approaches. The within-day CV decreased in all three channels, indicating more consistent plot-level RGB observations under the conditions studied. Tests across environments and with independent reflectance and agronomic measurements are needed to assess the wider use of this approach.

## Figures and Tables

**Table 1:** Main experimental equipment and models.

Equipment/ Instrument	Model	Manufacturer	Main Specifications
Multispectral Drone	DJI M3M	DJI, Shenzhen, China	One 20-megapixel visible light camera and four 5-megapixel multispectral cameras (green, red, red edge, and near-infrared).
Illuminance Meter	CEM AS823	SMART SENSOR, Dongguan, China	Output voltage: 0–5 V; supply voltage: 5–24 V; Measuring range: 0–20,000 lux.
Solar Altitude Angle Software	HEAVENS ABOVE	Heavens-Above GmbH, Munich, Germany	Capable of real-time observation of solar altitude angle, sunrise and sunset times, among other parameters.
White Reference Panel	Labsphere	Labsphere, Inc., North Sutton, NH, USA	Reflectance: 99% Size: 10 inches

**Table 2:** Extracted feature parameters.

Parameter Category	Parameter	Number of Parameters
Skewed parameter (SP)	*R_m_*_ean_, *R_m_*_edian_, *R_m_*_ode_, *R_s_*_kewness_, *R_k_*_urtosis,_ *G_m_*_ean_, *G_m_*_edian_, *G_m_*_ode_, *G_s_*_kewness_, *G_k_*_urtosis_, *B_m_*_ean_, *B_m_*_edian_, *B_m_*_ode_, *B_s_*_kewness_, *B_k_*_urtosis_, *Gray*_mean_, *Gray*_median_, *Gray*_mode_, *Gray*_skewness_, *Gray*_kurtosis_	20
Hue, Saturation, Value (HSV)	*H*_mean_, *S*_mean_, *V*_mean_	3
Lightness, a-axis, b-axis (Lab)	*L*_mean_, *a*_mean_, *b*_mean_	3
Blue Deficient Index (BDI)	*BDI*_min_, *BDI*_mean_, *BDI*_max_	3

Note: *R_m_*_ean_, *R_m_*_edian_, *R_m_*_ode_, *R_s_*_kewness_, and *R_k_*_urtosis_ are the mean, median, mode, skewness and kurtosis for the red (R) channel respectively; *G_m_*_ean_, *G_m_*_edian_, *G_m_*_ode_, *G_s_*_kewness_ and *G_k_*_urtosis_ are the mean, median, mode, skewness and kurtosis for the green (G) channel; *B_m_*_ean_, *B_m_*_edian_, *B_m_*_ode_, *B_s_*_kewness_ and *B_k_*_urtosis_ are the mean, median, mode, skewness and kurtosis for the blue (B) channel; and *Gray*_mean_, *Gray*_median_, *Gray*_mode_, *Gray*_skewness_ and *Gray*_kurtosis_ are the mean, median, mode, skewness and kurtosis for the grayscale image. *H*_mean_, *S*_mean_ and *V*_mean_ are the mean for the hue, saturation and value of the image. *L*_mean_, *a*_mean_, and *b*_mean_ represent the mean values of the lightness (L), red–green opponent (a), and yellow–blue opponent (b) channels in the CIE Lab colour space. *BDI*_min_, *BDI*_mean_, *BDI*_max_ are the minimum, mean, maximum for the BDI image.

**Table 3:** Feature-input configurations.

Input Configuration	Feature Composition	Number of Features	Prediction Target
Canopy	SP; HSV; Lab;	26	Rc/Gc/Bc
Canopy_BDI	SP; HSV; Lab; BDI	29	Rc/Gc/Bc
Fullgrade_flat	SP; HSV; Lab;	14 × 26	Rc/Gc/Bc
Fullgrade_noflat	SP; HSV; Lab;	[14, 26]	Rc/Gc/Bc

**Table 4:** Performance of the best models under different feature input configurations.

Input Configurations	Best ValLoss	Best Test R^2^	Test R^2^_ave_	Test RMSE_ave_	Test MAE_ave_
Canopy	BPNN	BPNN	0.8617	0.0224	0.0171
Canopy_BDI	BPNN	1D-CNN	0.8679	0.0221	0.0169
Fullgrade_flat	BPNN	KAN	0.8288	0.025	0.0189
Fullgrade_noflat	1D-CNN	1D-CNN	0.8773	0.0213	0.0161

**Table 5:** Performance of the best models under different feature input configurations.

Channel	Mean R^2^	Standard Deviation R^2^
Rc	0.9024	0.0194
Gc	0.8008	0.0685
Bc	0.8736	0.0371

**Table 6:** Performance comparison of different correction methods.

Method/Category	Channel	RMSE (Mean ± SD)	MAE (Mean ± SD)	CV (%) (Mean ± SD)
Original RGB	R	—	—	9.3862 ± 3.1721
	G	—	—	7.5191 ± 2.8201
	B	—	—	10.8073 ± 4.6157
Ours	R	0.0207 ± 0.0083	0.0169 ± 0.0078	4.4026 ± 1.2084
	G	0.0172 ± 0.0057	0.0142 ± 0.0053	2.7932 ± 0.7943
	B	0.0144 ± 0.0061	0.0121 ± 0.0056	4.6493 ± 1.6854
BDI-Otsu corrected	R	0.1018 ± 0.0163	0.0943 ± 0.0148	7.9805 ± 3.1373
	G	0.1095 ± 0.0279	0.1006 ± 0.0229	7.0606 ± 4.0191
	B	0.0387 ± 0.0126	0.0340 ± 0.0115	10.3963 ± 4.4360
RGB-Y-Otsu corrected	R	0.1694 ± 0.0300	0.1626 ± 0.0291	8.5938 ± 2.7888
	G	0.1931 ± 0.0320	0.1859 ± 0.0295	7.4704 ± 3.3939
	B	0.0838 ± 0.0327	0.0784 ± 0.0319	9.7929 ± 3.8170
SDI local corrected	R	0.2015 ± 0.0331	0.1949 ± 0.0326	8.6031 ± 2.9476
	G	0.2217 ± 0.0389	0.2147 ± 0.0367	7.5967 ± 3.3569
	B	0.1526 ± 0.0262	0.1473 ± 0.0249	10.4913 ± 3.3725

## Data Availability

The data will be shared upon request.

## Abbreviations

UAV	Unmanned aerial vehicle
RGB	Red–green–blue
HSV	Hue–saturation–value
BDI	Blue deficient index
SP	Skewed parameters
MLP	Multilayer perceptron
KAN	Kolmogorov–Arnold network
BPNN	Back-propagation neural network
LSTM	Long short-term memory network
1D-CNN	One-dimensional convolutional neural network
SHAP	Shapley additive explanations
RMSE	Root mean square error
MAE	Mean absolute error
R^2^	Coefficient of determination
CV	Coefficient of variation
